# *Ophiopogon japonicus* Root Extract Attenuates Obesity-Induced Muscle Atrophy Through Regulation of the PI3K-AKT-mTOR/FoxO3a Signaling Pathway and Lipid Metabolism in Mice and C2C12 Myotubes

**DOI:** 10.3390/nu17243946

**Published:** 2025-12-17

**Authors:** Yang Wang, Haifeng Shao, Chenzi Lyu, Kyung Hee Park, Tran Khoa Nguyen, In Jun Yang, Hyo Won Jung, Yong-Ki Park

**Affiliations:** 1 Department of Herbology, College of Korean Medicine, Dongguk University, Gyeongju 38066, Republic of Korea; wy1997ere@163.com (Y.W.); shf326904@163.com (H.S.); lcz18435166779@163.com (C.L.); 2024126916a@gmail.com (K.H.P.); yongki@dongguk.ac.kr (Y.-K.P.); 2 Department of Physiology, College of Korean Medicine, Dongguk University, Gyeongju 38066, Republic of Korea; trannguyen053@gmail.com (T.K.N.); injuny@gmail.com (I.J.Y.)

**Keywords:** *Ophiopogon japonicus*, muscle atrophy, lipid metabolism, obesity, PI3K-AKT-mTOR/FoxO3a

## Abstract

**Background:** Obesity-associated skeletal muscle atrophy is characterized by reduced muscle mass with excessive adipose accumulation, and there is no approved pharmacological therapy targeting both muscle anabolism and lipid metabolism. The root part of *Ophiopogon japonicus* (OJ), an edible traditional medicine (Liriopis seu Ophiopogonis Tuber), exhibits anti-diabetic, anti-inflammatory, and cardioprotective properties, yet its impact on obesity-associated muscle atrophy remains unknown. **Methods:** This study investigated the therapeutic potential and mechanisms of OJ extract against muscle atrophy in high-fat diet (HFD)-induced obesity mice and palmitate (PA)-stimulated C2C12 myotubes. **Results:** In obese mice, the administration of OJ extract inhibited muscle loss, improved muscle strength, and attenuated hepatic steatosis and dyslipidemia. Furthermore, OJ treatment restored myotube diameter, increased the expression of MyHC and Myogenin, and suppressed the expression of Atrogin-1 and MuRF1 in C2C12 myotubes. At the molecular level, OJ extract activated the PI3K-AKT-mTOR/FoxO3a signaling pathway and reprogrammed lipid metabolism in gastrocnemius tissues and myotubes. **Conclusions:** OJ extract alleviates obesity-induced muscle atrophy through regulation of the PI3K-AKT-mTOR/FoxO3a signaling pathway and lipid metabolism in muscle, indicating its potential as a natural therapeutic agent for obesity-associated muscle atrophy.

## 1. Introduction

Obesity has emerged as a global health crisis, with its associated muscle atrophy representing a metabolic myopathy characterized by reduced muscle mass and excessive adipose tissue accumulation [1,2]. With the global rise in obesity prevalence and the acceleration of population aging, obesity-associated muscle atrophy has become an urgent public health issue [3]. Epidemiological studies have reported that its global prevalence is approximately 11% among individuals over 60 years of age and reaches 16.7% in those over 80 years [4,5]. This condition involves progressive loss of muscle mass and function driven by lipid metabolic disorders, chronic inflammation, insulin resistance, and mitochondrial dysfunction, ultimately impairing mobility and metabolic health [6].

Current management of obesity-associated muscle atrophy primarily relies on lifestyle interventions, including resistance training and high-protein diets, often combined with insulin-sensitizing agents such as metformin [7]. Although these strategies can alleviate symptoms, their long-term adherence and efficacy are limited. Moreover, there are currently no approved pharmacological therapies that can simultaneously promote muscle growth and reduce adiposity. Therefore, exploring effective and safe nutritional interventions has become increasingly important.

In recent years, growing evidence has shown that various botanical extracts can modulate glucose and insulin homeostasis, thereby exerting protective effects against metabolic disorders [8]. Numerous plant-derived ingredients have been reported to enhance insulin sensitivity and promote glucose uptake primarily through activation of the IRS-1/PI3K/Akt signaling pathway. For example, *Cucumis prophetarum* extract improves insulin responsiveness by activating the IRS-1/Akt pathway [9]. In addition, ginsenosides from *Panax ginseng*, berberine from *Coptis chinensis*, and polyphenols from *Camellia sinensis* ameliorate insulin resistance by restoring the PI3K/Akt signaling activity in various diabetic models [10]. These findings highlight the capacity of natural products to act as metabolic regulators through the core insulin signaling cascade, a pathway closely associated with muscle protein synthesis and glucose utilization.

The root of *Ophiopogon japonicus* (Thunb.) Ker-Gawler (Liriopis seu Ophiopogonis Tuber, Liliaceae) is a perennial herb widely distributed across East Asia and has been used as a “yin-nourishing” essential herb in traditional Chinese and Korean medicine for millennia [11,12]. Pharmacological investigations have validated its broad therapeutic potential, including anti-diabetic, anti-inflammatory, and cardioprotective effects, which are closely associated with its rich content of saponins, flavonoids, and polysaccharides [13,14,15]. Notably, O. japonicus, classified as a dual-use substance with both medicinal and edible value, was officially added to Chinese National Health Commission’s Catalogue of Food and Traditional Medicinal Substances in August 2024, thereby providing a strong policy basis for its application in preventive healthcare [16,17,18].

Although previous studies have demonstrated that OJ extract improved glucose metabolic disorders [19,20,21], its effects on obesity-associated muscle atrophy remain unclear. Therefore, this study systematically investigated the therapeutic efficacy and molecular mechanisms of OJ extract against obesity-induced muscle atrophy in mice and palmitate (PA)-induced atrophy in C2C12 myotubes.

## 2. Materials and Methods

### 2.1. Preparation of OJ Extract

The dried roots of *Ophiopogon japonicus* were obtained from Kwangmyungdang Co. (Ulsan, Republic of Korea), and their taxonomic identity was verified by Prof. Y.-K. Park from Dongguk University. A voucher specimen (No. 2025-W-02) has been archived in the university herbarium.

For extraction, 200 g of root powder underwent two successive cycles of boiling water reflux in 2 L of distilled water for 2 h each. The pooled extracts were concentrated at 60 °C using a vacuum rotary evaporator (Eyela Co., Ltd., Tokyo, Japan). Ethanol was then mixed into the concentrate until a 60% (*v*/*v*) solution was reached, and the mixture was allowed to precipitate overnight. The mixture was then subjected to suction filtration through filter paper and vacuum-concentrated again. Finally, the concentrated solution was lyophilized at −80 °C under 5 mTorr using a lyophilizer (IlShin Lab Co., Jeonju, Republic of Korea). The dried extract (yield, 56.4%) was ground into powder and stored at −20 °C for later use.

### 2.2. Preparation of Animal Model

A total of 30 male C57BL/6 mice (8 weeks) were obtained from Koatech Inc. (Busan, Republic of Korea). All animal experimental procedures were performed strictly following the Guide for the Welfare and Ethics of Laboratory Animals (The Ministry of Food and Drug Safety, Republic of Korea) and were approved by the Institutional Animal Care and Use Committee (IACUC) of Dongguk University (approval No. IACUC-2024-07). Mice were maintained in a controlled environment set at 22 ± 3 °C, with approximately 60% relative humidity and a 12 h light/12 h dark cycle, and were given unrestricted access to food and water. After a 7-day acclimation period, the animals were randomly allocated into two initial groups: Control group (CON, *n* = 6) received a standard chow diet providing 3.10 kcal/g (18% fat, 58% carbohydrate, 24% protein) and High-fat diet group (HFD, *n* = 24) was offered a high-fat formulation delivering 5.24 kcal/g (60% fat, 20% carbohydrate, 20% protein).

According to the Chinese Pharmacopoeia (CP), the approved human dosage of Maidong whole-water extract is 6–12 g for a 60 kg individual [20]. The mouse-equivalent dose was calculated using the body-surface-area conversion formula (Km factor = 9.01), corresponding to 1.8 g/kg. Given that the weight ratio of OJ extract was 56.4% of the whole crude drug mass, three experimental dose groups (high, medium, and low) were established for mice at 1.0 g/kg, 0.5 g/kg, and 0.25 g/kg, respectively.

Following 16 weeks of feeding, the HFD group was further randomly subdivided into four subgroups: Model group (MOD, *n* = 6), OJ-250 mg/kg group (OJ-low dose, OJL, *n* = 6), OJ-500 mg/kg group (OJ-medium dose, OJM, *n* = 6), and OJ-1000 mg/kg group (OJ-high dose, OJH, *n* = 6). OJ extract was administered orally to the treatment groups once daily for 6 weeks, while CON and MOD groups received equivalent volumes of distilled water via gavage. Body weight and grip strength were recorded weekly. After the 6-week administration period, the animals were fasted overnight, and the final dose of the OJ extract was administered 24 h before euthanasia. The mice were subsequently euthanized by inhalation of a mixed anesthetic gas containing 75% O_2_, 25% N_2_O, and 5% isoflurane for more than 3 min. Death was confirmed by the absence of heartbeat and thoracic movement, pallor of the mucous membranes, and lack of response to toe pinching. Immediately thereafter, whole blood (0.7–0.8 mL) was collected via cardiac puncture. The blood samples were allowed to clot at room temperature for 2 h and then centrifuged at 3500 rpm for 15 min at 4 °C, and the resulting supernatant was collected as serum. Adipose tissues, liver, and muscle samples were also harvested for subsequent analyses.

### 2.3. Measurement of Grip Strength

Grip strength was assessed with a digital force-measurement device (Jeongdo Bio & Plant Co., Ltd., Seoul, Republic of Korea). Briefly, the animals were positioned on the metal grid and allowed a brief period to familiarize themselves with the apparatus. After stabilization, investigators gently grasped the tail and pulled horizontally backward until the animal released its grip. Three measurements were performed per mouse, averaged, and the mean values were normalized to body weight for analysis.

### 2.4. Hanging Time Test

The animals were gently positioned on a wire mesh, allowing them to grasp the surface securely with all four limbs. The mesh was then turned upside down, and the latency to fall was monitored until the mouse released its hold from fatigue. The duration was normalized to body weight for analysis, with a maximum test duration of 3 min.

### 2.5. Glucose Tolerance and Insulin Resistance Tests

For oral glucose tolerance test (OGTT), mice were fasted for 12 h and then given a 20% (*w*/*v*) glucose solution by oral gavage at a dose of 2 g/kg. Tail-vein blood was obtained at 0, 30, 60, and 120 min, and glucose concentrations were determined using Accu-Chek Guide test strips (Roche Diabetes Care, Mannheim, Germany).

For insulin resistance test (ITT), the mice underwent a 4 h fasting period and were subsequently injected intraperitoneally with insulin at 0.75 U/kg. Blood glucose was tracked at 0, 15, 30, 60, and 120 min following insulin administration.

### 2.6. Histopathological Analysis

Gastrocnemius muscle and liver samples were harvested from mice and immersed in 4% paraformaldehyde for 24 h to achieve fixation. Following dehydration and paraffin embedding, tissues were sectioned into 4 μm thick slices and stained with hematoxylin-eosin (H&E). The stained slides were subsequently dehydrated through graded ethanol, cleared with xylene, and coverslipped using a neutral mounting medium. After microscopic examination and imaging, the cross-sectional area (CSA) of muscle fibers was analyzed with ImageJ software (version 1.52a, https://imagej.net/ij/, accessed on 1 May 2025). The severity of liver injury was evaluated according to the criteria outlined in Table S1.

### 2.7. Analysis of Serological Parameters

Serum samples were analyzed for the levels of aspartate aminotransferase (AST), alanine aminotransferase (ALT), total cholesterol (TCHO), triglycerides (TG), high-density lipoprotein-cholesterol (HDL-C), and low-density lipoprotein-cholesterol (LDL-C) using an automatic clinical chemistry analyzer (FDC7000i, Fujifilm Co., Tokyo, Japan).

### 2.8. C2C12 Cell Cultures and Treatment

The C2C12 mouse myoblasts (CRL-1772, ATCC, Manassas, VA, USA) were cultured in a humidified incubator at 37 °C with 5% CO_2_. The growth medium consisted of DMEM supplemented with 10% fetal bovine serum (FBS, Merck Millipore, Burlington, MA, USA) and 1% penicillin/streptomycin (P/S, Merck Millipore). Upon reaching roughly 90% confluence, the medium was replaced with differentiation medium containing 2% horse serum (HS, Merck Millipore) and 1% P/S, which was renewed every other day for 4 days from myoblasts to myotubes.

To model lipid-induced myotube atrophy, differentiated cells were exposed to palmitic acid (0.25, 0.5, 0.75, or 1 mM) for 48 h to identify an appropriate stimulation concentration. Next, the effect of OJ extract on PA-induced atrophic changes was then evaluated. Myotubes were treated with OJ extract at 0.25, 0.5, or 1 mg/mL with or without 0.5 mM PA for 48 h. In the control group, myotubes were treated with the vehicle solvent (1% bovine serum albumin, BSA).

### 2.9. Cell Viability Analysis

C2C12 myoblasts (1 × 10^5^ cells/mL) were seeded into 24-well plates and allowed to differentiate for 4 days to form myotubes. After differentiation, the cells were exposed to OJ extract at 0.25, 0.5, or 1 mg/mL, either alone or together with 0.5 mM PA, for a 48 h incubation. Subsequently, 50 μL of EZ-Cytox reagent (EZ-500, DoGenBio, Seoul, Republic of Korea) was added to each well, and the plates were maintained at 37 °C for an additional 2 h. Following the reaction, the absorbance at 450 nm was measured with a Biochrom ASYS UVM 340 Plate Reader (ASYS Hitech GmbH, Eugendorf, Austria).

### 2.10. Immunocytochemistry

The C2C12 myoblasts were seeded in 12-well culture plates with ThermanoxTM plastic coverslips (Nunc, Thermo Fisher Scientific, Waltham, MA, USA). Upon reaching >90% confluence, myoblasts were differentiated into myotubes by replacing medium for 4 days, and then treated with OJ extract with or without PA for 48 h.

After aspirating the culture medium, cells were rinsed three times with 1 × PBS and fixed in 4% paraformaldehyde for 30 min at room temperature (RT). Permeabilization was performed with 0.5% Triton X-100 (Sigma-Aldrich, Saint Louis, MO, USA) for 5 min, followed by blocking with 5% BSA for 2 h. The cells were incubated with primary anti-MHC antibody (sc-376157, Santa Cruz Biotechnology Inc., Paso Robles, CA, USA) overnight at 4 °C. Subsequently, the cells were incubated with fluorescent secondary antibody (A11001, Thermo Fisher Scientific) for 2 h at RT in the dark, followed by DAPI staining for 5 min. The coverslips were mounted using anti-fade reagent and visualized under a fluorescence microscope (Leica DM2500, Leica Microsystems, Wetzlar, Germany).

### 2.11. Oil Red O Staining

Differentiated C2C12 myotubes were exposed to PA for 24 h, and lipid accumulation in the cytoplasm was assessed using an Oil Red O staining kit (G1262, Beijing Solarbio Science & Technology Co., Beijing, China). After removal of the culture medium, the cells were fixed in 4% paraformaldehyde for 30 min at RT and then incubated with freshly prepared Oil Red O solution for 20 min. Excess dye was removed, followed by a brief rinse with 60% isopropanol for 30 s and thorough washing with distilled water until no residual staining remained. Subsequently, Mayer’s hematoxylin solution was added for 2 min to counterstain of nuclei. The coverslips were mounted in distilled water, and stained cells were visualized under a light microscope. Lipid accumulation was quantified semi-quantitatively using ImageJ software.

### 2.12. Quantitative Real-Time PCR Analysis

RNA was isolated from C2C12 myotubes and gastrocnemius muscle using TRIzol reagent (15596026, Invitrogen, Waltham, MA, USA) at RT in accordance with the manufacturer’s instructions. The quantity of RNA was assessed with a spectrophotometer (NBL-C-240504, MicroDigital Inc., Seoul, Republic of Korea). cDNA was synthesized from the extracted RNA with the ReverTra Ace™ qPCR RT Master Mix kit (FSQ-201, TOYOBO Co., Ltd., Osaka, Japan). Quantitative PCR was performed using the SYBR Green PCR kit on an iCycler iQ™ Real-Time PCR Detection System (Bio-Rad Laboratories, Hercules, CA, USA) to assess mRNA expression. Relative expression levels were calculated by the 2^−ΔΔCt^ method with GAPDH as the reference gene. Primer sequences for SYBR Green reactions are provided in Table S2.

### 2.13. Western Blotting Analysis

Protein was isolated from C2C12 myotubes and gastrocnemius muscle tissues using ice-cold RIPA lysis buffer (89901, Thermo Fisher Scientific) supplemented with protease/phosphatase inhibitors. For immunoblotting, 30 µg of protein was resolved on 8% or 10% sodium dodecyl sulfate-polyacrylamide gel electrophoresis (SDS-PAGE) gels and transferred to nitrocellulose membranes (Cytiva, New York, NY, USA). The membranes were incubated in 5% non-fat dry milk for 2 h at RT to block nonspecific binding and subsequently incubated overnight at 4 °C with the respective primary antibodies, including MyHC (sc-376157, Santa Cruz), Myogenin (sc-52903), MuRF1 (bs-2539R, Bioss Antibodies, Woburn, MA, USA), Atrogin-1 (PA5-91959, Thermo Fisher Scientific), *p*-FoxO3a (PA5-36816), FoxO3a (PA5-27145), SREBP-1c (PA1-337), CPT1b (PA5-79065), mTOR (2972s, Cell Signaling, Danvers, MA, USA), *p*-mTOR (5536s), AKT (9272s), *p*-AKT (9271s), PI3K (4292s), *p*-PI3K (4228s), β-Actin (A5316, Sigma-Aldrich), and GAPDH (PA0018). After washing, membranes were incubated with horseradish peroxidase (HRP)-conjugated anti-mouse or anti-rabbit IgG secondary antibodies (Bio-Rad Laboratories) at RT for 2 h. Protein bands were visualized using the ChemiDoc XRS Imaging System (Bio-Rad Laboratories), and densitometric analysis was performed with ImageJ software for quantitative evaluation.

### 2.14. Ultra-High Performance Liquid Chromatography–Quadrupole Time-of-Flight Mass Spectrometry (UHPLC-Q-TOF-MS/MS)

The UHPLC-Q-TOF-MS/MS technique was employed to analyze the compounds in the OJ extract, and the experimental details are described below. Chromatographic separation was carried out on an ACQUITY UPLC HSS T3 column (2.1 × 100 mm, 1.8 µm). The binary mobile phase included acetonitrile (solvent A) and 0.1% formic acid in water (solvent B). The column was maintained at 35 °C with a flow rate of 0.3 mL/min, and 2 μL of sample was injected for each analysis. The gradient elution program was applied as follows: 0–70 min, 0–50% A; 70–85 min, 50–95% A; 85–90 min, 95% A; 90–96 min, 0% A. A high-resolution Q-TOF mass spectrometer (Agilent 6546, Santa Clara, CA, USA) was employed for mass spectrometric analysis. The ESI source settings were as follows: nozzle voltage, +4.0/−3.5 kV; sheath gas at 350 °C and 11 L/min, and drying gas at 8 L/min at 325 °C. Full-scan MS spectra were recorded from *m*/*z* 100 to 1700, and MS/MS fragmentation spectra were obtained using stepped collision energies of 10, 20, and 40 eV.

### 2.15. Network Pharmacology Analysis

Active components of *Ophiopogon japonicus* (OJ) and their potential targets were obtained from The Encyclopedia of Traditional Chinese Medicine (ETCM) and Bioinformatics Annotation daTabase for Molecular mechANism of Traditional Chinese Medicine (BATMAN-TCM). Targets associated with obesity-induced muscle atrophy (OIMA) were acquired from GeneCards, OMIM, and DisGeNET databases. Overlapping targets among these databases were excluded. Protein–protein interaction (PPI) information for the merged targets was obtained from the STRING database with species parameter set to “Homo sapiens”. High-confidence protein interactions were filtered at a minimum required interaction score >10. The resulting interaction data were analyzed in Cytoscape (version 3.10.2) for core target identification using the CentiScaPe 2.2 plug-in and PPI network visualization. Gene Ontology (GO) annotation and Kyoto Encyclopedia of Genes and Genomes (KEGG) pathway enrichment analyses of common targets were performed via Metascape. Finally, a comprehensive regulatory network integrating active compounds, targets, and pathways was constructed using Cytoscape to elucidate OJ-OIMA interactions.

### 2.16. Statistical Analysis

Quantitative data are expressed as the mean ± SD, derived from at least three independent experiments. Statistical processing was conducted using GraphPad Prism 8.0. Differences among groups were analyzed through one-way ANOVA, followed by Tukey’s post hoc test for multiple comparisons. A *p*-value below 0.05 was considered indicative of statistical significance.

## 3. Results

### 3.1. OJ Extract Ameliorated Obesity-Induced Muscle Atrophy in Mice

**Figure 1:**
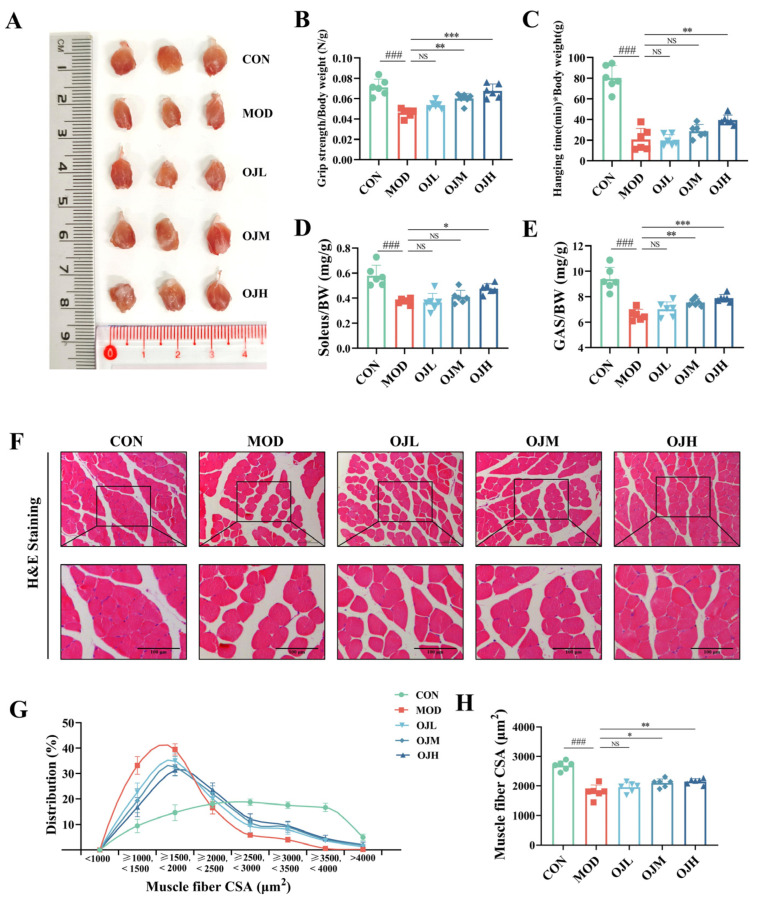
Effects of OJ extract on muscle strength and mass in obesity-induced muscle atrophy mice. (**A**) Representative images of GAS tissues from each group. (**B**) Grip strength assessment. (**C**) Hanging time test. (**D**) The ratio of soleus mass (mg) normalized to body weight (g). (**E**) The ratio of GAS mass (mg) normalized to body weight (g). (**F**) H&E-stained cross-sections of GAS. (**G**) Distribution of myofiber cross-sectional area in the GAS. (**H**) Mean cross-sectional area of GAS across groups. All data are presented as the mean ± SD (*n* = 6). The *p* values were defined as follows: ### *p* < 0.001 vs. CON; * *p* < 0.05, ** *p* < 0.01 and *** *p* < 0.001 vs. MOD; NS = not significant (*p* ≥ 0.05).

To investigate the potential regulatory effects of OJ extract on muscle atrophy, we established a mouse model of obesity-induced muscle atrophy through 16 weeks of HFD feeding. Compared with the CON group, obese mice showed markedly decreased grip strength and shorter hanging time (*p* < 0.01, Figure 1B,C), indicating impaired muscle function. However, OJ extract treatment effectively improved muscle performance. Muscle mass analysis revealed that obesity markedly decreased the relative weights of soleus and gastrocnemius (Figure 1A,D,E). OJ extract administration significantly attenuated these losses, particularly in the OJH groups (*p* < 0.05).

H&E staining further revealed pronounced muscle fiber atrophy in MOD mice, as evidenced by reduced cross-sectional area (CSA) and irregular morphology (Figure 1F). Quantitative analysis showed a leftward shift of the myofiber CSA distribution in the MOD group, whereas OJ extract treatment shifted the distribution rightward toward larger fibers (Figure 1G). Consistently, the mean CSA of muscle fibers was significantly reduced in MOD mice (*p* < 0.001), but was restored by OJ extract (*p* < 0.05, Figure 1H).

These results indicate that OJ extract ameliorates obesity-induced muscle atrophy by improving muscle strength, preserving muscle mass, and restoring myofiber size.

### 3.2. OJ Extract Improved Glucose Tolerance, Insulin Resistance, and Dyslipidemia in Obesity-Induced Muscle Atrophy Mice

**Figure 2:**
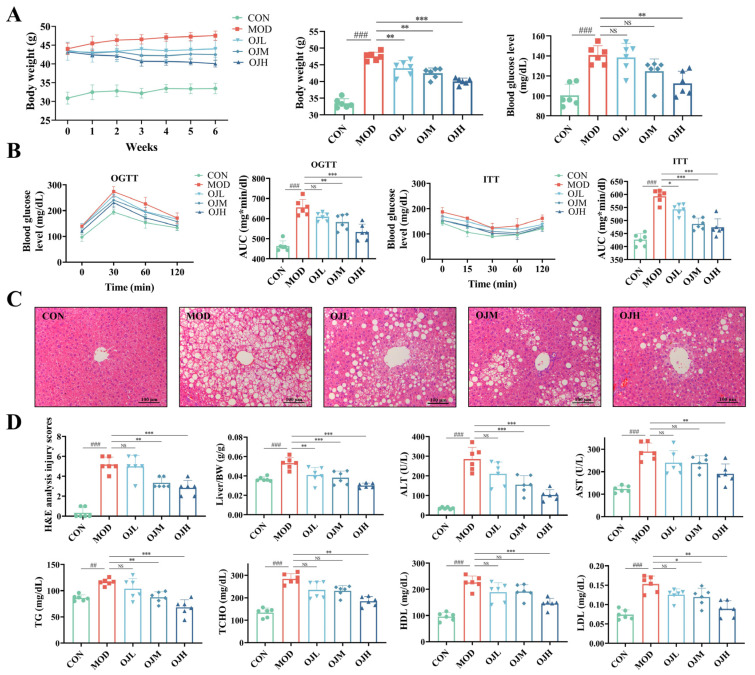
Impacts of OJ extract on the glucose tolerance, insulin resistance, and dyslipidemia in obesity-induced muscle atrophy mice. (**A**) Variations in body weight during the treatment period, terminal body weight records, and fasting blood glucose concentrations. (**B**) Oral glucose tolerance test (OGTT), insulin tolerance test (ITT), and the corresponding areas under the curve (AUC). (**C**) H&E staining of liver tissues. (**D**) Liver injury scores, liver-to-body weight ratios, and serum biochemical parameters of ALT, AST, TG, TCHO, HDL-C, and LDL-C. All data are presented as the mean ± SD (*n* = 6). The *p* values were defined as follows: ## *p* < 0.01 and ### *p* < 0.001 vs. CON; * *p* < 0.05, ** *p* < 0.01 and *** *p* < 0.001 vs. MOD; NS = not significant (*p* ≥ 0.05).

We further investigated the effects of OJ extract on the glucose tolerance, insulin resistance, and dyslipidemia in obesity-induced muscle atrophy mice. After 16 weeks of HFD feeding, body weight was obviously higher in the MOD group than in the CON group (Figure 2A). Following 6 weeks of OJ administration, both BW and fasting blood glucose (FBG) levels were significantly reduced in the OJH group compared to the MOD group (*p* < 0.01, Figure 2A). Notably, food intake did not differ significantly between OJ-treated and MOD groups, suggesting that the beneficial effects of OJ extract are independent of alterations in food intake (Figure S1A). Furthermore, OGTT and ITT results demonstrated that OJ extract significantly improved glucose intolerance and insulin resistance in obese mice (*p* < 0.05, Figure 2B).

H&E staining of liver tissues revealed regularly arranged hepatocytes with normal architecture, displaying normal morphology without detectable pathological alterations in the CON group. Conversely, the MOD group exhibited extensive diffuse hepatic steatosis, inflammatory cell infiltration, and hepatocellular ballooning degeneration (Figure 2C). Notably, supplementation with OJ extract attenuated these pathological alterations, resulting in significant decreases of histological scoring including hepatic inflammation and lipid accumulation (*p* < 0.01, Figure 2D).

To assess systemic lipid deposition, we measured the relative weights of liver and adipose tissues. In the OJH group, the relative weights of the liver, inguinal white adipose tissue (iWAT), and epididymal white adipose tissue (eWAT) were markedly decreased (*p* < 0.05, Figure 2D and Figure S1B,C), while no significant difference was observed in brown adipose tissue (BAT) (Figure S1D).

Serological analysis showed that the MOD group had significantly higher levels of ALT, AST, TG, TCHO, HDL-cholesterol, and LDL-cholesterol than the CON group, while administration of OJ extract significantly reduced these elevations (*p* < 0.05, Figure 2D).

These results indicate that OJ extract exerts protective effects in obesity-induced muscle atrophy mice by reducing body weight, fasting blood glucose, hepatic steatosis, and systemic lipid accumulation.

### 3.3. OJ Extract Modulated the PI3K-AKT-mTOR/FoxO3a Signaling and Lipid Metabolism in Muscle Tissues of Obesity-Induced Muscle Atrophy Mice

**Figure 3:**
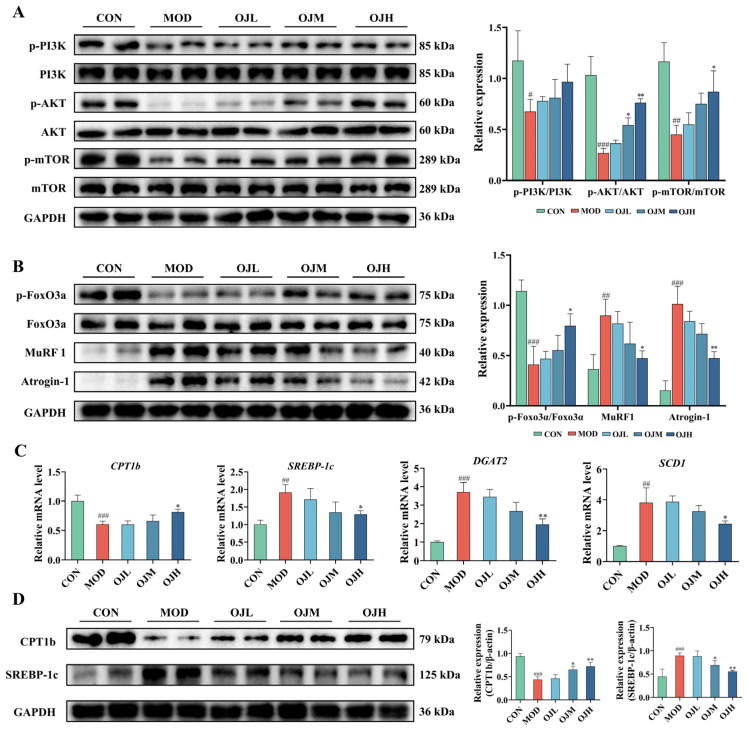
Effects of OJ extract on regulation of the PI3K-AKT-mTOR/FoxO3a signaling and lipid metabolism in muscle tissues of obesity-induced muscle atrophy mice. (**A**) Quantification of phosphorylation ratios for PI3K, AKT, mTOR, and FoxO3a. (**B**) Expression levels of MuRF1 and Atrogin-1. (**C**) Relative mRNA levels of *CPT1b*, *SREBP-1c*, *DGAT2*, and *SCD1*. (**D**) Protein levels of CPT1b and SREBP-1c. All data are presented as the mean ± SD (*n* = 6). The *p* values were defined as follows: # *p* < 0.05, ## *p* < 0.01 and ### *p* < 0.001 vs. CON; * *p* < 0.05 and ** *p* < 0.01 vs. MOD.

To elucidate the mechanism by which OJ extract ameliorates muscle atrophy, we evaluated the PI3K-AKT-mTOR/FoxO3a signaling pathway in muscle tissues of obesity-induced muscle atrophy mice. Results showed phosphorylation levels of PI3K, AKT, mTOR, and FoxO3a were significantly reduced in the MOD group compared with the CON group (*p* < 0.05, Figure 3A,B). However, the administration of OJ extract at high dose (OJH) significantly elevated the phosphorylation of AKT, mTOR, and FoxO3a in gastrocnemius tissues compared with the MOD group (*p* < 0.05). Furthermore, the expression of muscle atrophy markers, MuRF1 and Atrogin-1, were markedly increased in the MOD group (*p* < 0.01), but significantly suppressed by OJH treatment (*p* < 0.05 for MuRF1, *p* < 0.01 for Atrogin-1, Figure 3B).

RT-qPCR analysis also revealed that the mRNA levels of *CPT1b* were decreased in the MOD group but significantly elevated in the OJH group (*p* < 0.05, Figure 3C). Conversely, the mRNA expression levels of *SREBP-1c*, *DGAT2*, and *SCD1* were significantly increased in the MOD group but reduced by OJ extract treatment (*p* < 0.05, Figure 3C).

Western blotting further showed that MOD group exhibited reduced CPT1b and elevated SREBP-1c protein levels, these alterations were reversed by OJH treatment (*p* < 0.01, Figure 3D).

These results suggest that OJ alleviates obesity-induced muscle atrophy by activating the PI3K-AKT-mTOR/FoxO3a pathway, upregulating CPT1b, and downregulating SREBP-1c, thereby enhancing protein synthesis pathways, suppressing proteolysis, and improving lipid metabolism in skeletal muscle.

### 3.4. OJ Extract Alleviated PA-Induced Atrophy in C2C12 Myotubes

**Figure 4:**
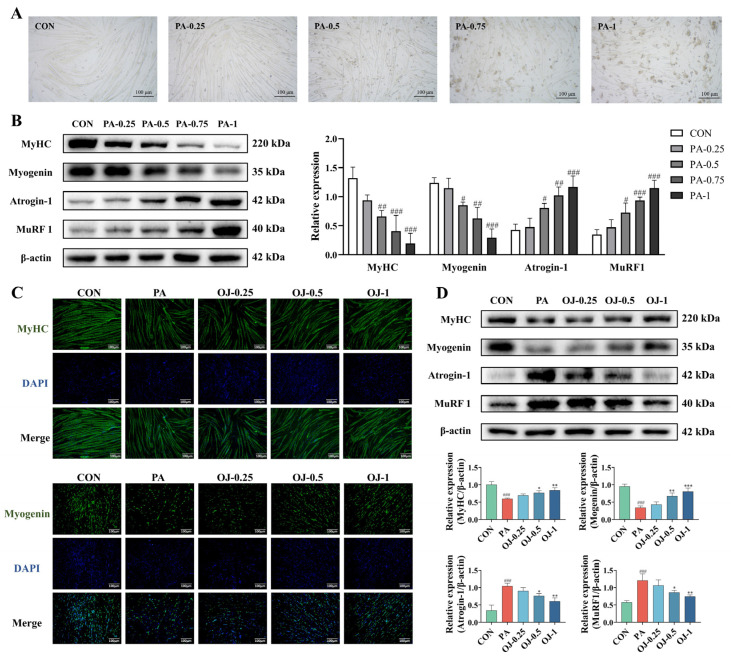
Effects of OJ extract on PA-induced atrophy in C2C12 myotubes. (**A**) Morphological change in C2C12 myotubes after treatment of PA at different concentrations for 48 h. (**B**) Protein expression of MyHC, Myogenin, Atrogin-1, and MuRF1 in C2C12 myotubes with PA treatment. (**C**) Representative images of MyHC and Myogenin immunofluorescence in C2C12 myotubes (Scale bar = 100 μm). (**D**) Expression of MyHC, Myogenin, Atrogin-1, and MuRF1 in C2C12 myotubes after treatment of OJ extract at different concentrations with PA at 0.5 mM. All data are presented as the mean ± SD (*n* = 3). The *p* values were defined as follows: # *p* < 0.05, ## *p* < 0.01 and ### *p* < 0.001 vs. CON; * *p* < 0.05, ** *p* < 0.01 and *** *p* < 0.001 vs. PA.

After differentiation, C2C12 myoblasts exhibited typical morphology of myotubes with elongated and multinucleated structures, parallel alignment, and visible sarcomeric striations (Figure 4A). In PA-treated myotubes, a decrease in cell viability was observed, with values of 98.9% at 0.25 mM, 92.49% at 0.5 mM, 83.38% at 0.75 mM, and 69.74% at 1 mM (*p* < 0.001, Figure S2). Moreover, PA treatment significantly reduced the expression of MyHC and Myogenin in C2C12 myotubes, while increasing the expression of Atrogin-1 and MuRF1 (*p* < 0.05, Figure 4B).

C2C12 myotubes were co-treated with OJ at various concentrations and 0.5 mM PA for 48 h, resulting in a slight but non-significant decrease in cell viability (Figure S2B). Immunofluorescence analysis revealed that OJ extract treatment significantly attenuated PA-induced downregulation of MyHC and Myogenin in C2C12 myotubes (*p* < 0.05, Figure 4C and Figure S2C,D). Consistently, Western blot analysis showed that PA exposure significantly reduced MyHC and Myogenin protein levels and increased Atrogin-1 and MuRF1 expression, whereas OJ extract treatment reversed these alterations (*p* < 0.001, Figure 4D).

These results suggest that OJ extract alleviates PA-induced muscle atrophy by elevating MyHC and Myogenin expression while reducing Atrogin-1 and MuRF1 levels.

### 3.5. OJ Extract Regulated the PI3K-AKT-mTOR/FoxO3a Signaling in PA-Stimulated C2C12 Myotubes

**Figure 5:**
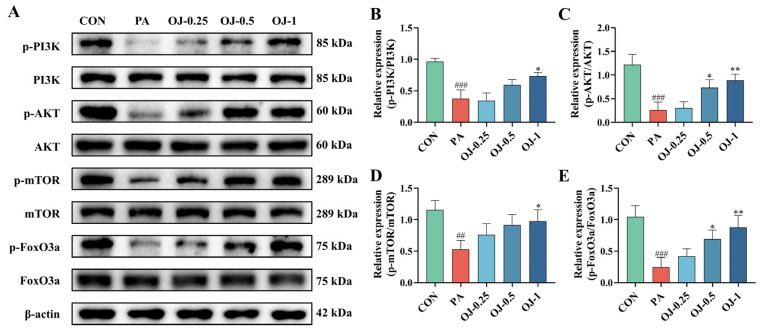
Effects of OJ extract on regulation of the PI3K-AKT-mTOR/FoxO3a pathway in PA-stimulated C2C12 myotubes. (**A**) The representative band of each target protein. (**B**–**E**) Quantification of phosphorylation ratios for PI3K, AKT, mTOR, and FoxO3a. All data are presented as the mean ± SD (*n* = 3). The *p* values were defined as follows: ## *p* < 0.01 and ### *p* < 0.001 vs. CON; * *p* < 0.05 and ** *p* < 0.01 vs. PA.

To identify the mechanism of OJ extract on muscle atrophy, we investigated the phosphorylation of the PI3K-AKT-mTOR/FoxO3a signaling pathway, which plays a crucial role in regulating muscle atrophy. As shown in Figure 5, the phosphorylation levels of PI3K (*p* < 0.001), AKT (*p* < 0.001), mTOR (*p* < 0.01), and FoxO3a (*p* < 0.001) were significantly lower in PA-stimulated C2C12 myotubes than in the CON group (Figure 5B–E). However, OJ treatment significantly enhanced the phosphorylation of PI3K, AKT, mTOR, and FoxO3a compared with the PA group (*p* < 0.05, Figure 5B–E). These findings suggest that OJ extract alleviates PA-induced atrophy by regulating the PI3K-AKT-mTOR/FoxO3a signaling pathway in C2C12 myotubes.

### 3.6. OJ Extract Improved Lipid Metabolism in PA-Stimulated C2C12 Myotubes

**Figure 6:**
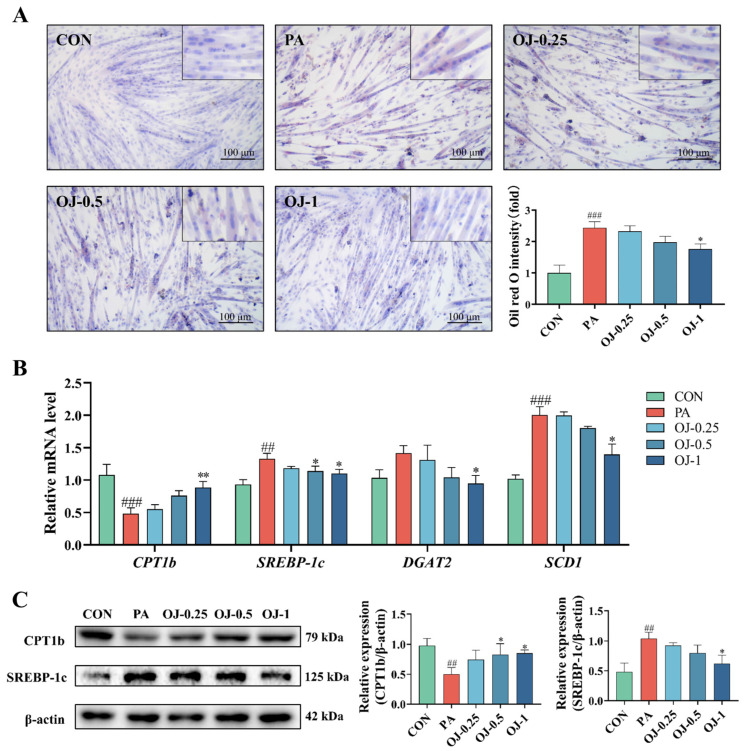
Effects of OJ extract on lipid metabolism in PA-stimulated C2C12 myotubes. (**A**) Representative images of Oil Red O staining (Scale bar = 100 μm) and relative intensity. (**B**) Relative mRNA expression levels of *CPT1b*, *SREBP-1c*, *DGAT2*, and *SCD1* in C2C12 myotubes. (**C**) Expression levels of CPT1b and SREBP-1c proteins in C2C12 myotubes. All data are presented as the mean ± SD (*n* = 3). The *p* values were defined as follows: ## *p* < 0.01 and ### *p* < 0.001 vs. CON; * *p* < 0.05 and ** *p* < 0.01 vs. PA.

To investigate the effects of OJ extract on the regulation of lipid metabolism in muscle atrophy, we investigated lipid accumulation and the expression of lipid oxidation and synthesis-regulatory genes in PA-stimulated C2C12 myotubes. The results of Oil Red O staining revealed red lipid droplets in the cytoplasm of cells in the PA group (Figure 6A). Compared with the PA group, OJ extract treatment significantly reduced the lipid accumulation in the cells (*p* < 0.05). In RT-qPCR analysis, PA stimulation in C2C12 myotubes significantly upregulated the mRNA levels of *CPT1b*, *SREBP-1c*, *DGAT2*, and *SCD1* compared with the CON group. Meanwhile, OJ extract treatment significantly reduced the mRNA levels of *CPT1b*, *SREBP-1c*, *DGAT2*, and *SCD1* (*p* < 0.05, Figure 6B). Compared with the PA group, OJ extract treatment significantly elevated the protein expression levels of CPT1b, and reduced the protein expression levels of SREBP-1c (*p* < 0.05, Figure 6C). These results indicate that OJ extract ameliorates PA-induced lipid accumulation and oxidation by modulating the lipid metabolism-regulatory genes in C2C12 myotubes.

### 3.7. UHPLC-Q-TOF-MS/MS Analysis of OJ Extract

**Figure 7:**
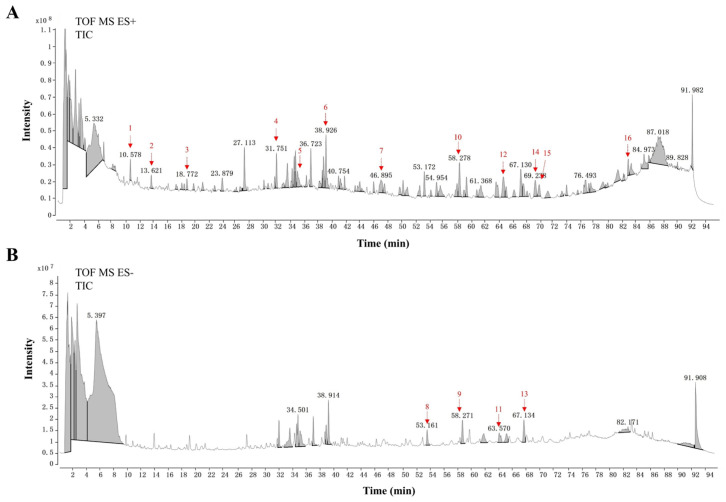
UHPLC–Q-TOF–MS/MS characterization of the OJ extract. (**A**) Total ion chromatographic profile of OJ extract acquired under positive electrospray ionization conditions. (**B**) Total ion chromatographic profile of OJ extract obtained under negative electrospray ionization mode.

The UHPLC-Q-TOF-MS/MS was conducted to detect the compounds in the OJ extract, and total ion chromatograms were obtained in both positive (Figure 7A) and negative (Figure 7B) electrospray ionization modes. The chemical composition of the OJ extract was further analyzed and identified using Qualitative Analysis 10.0 software in combination with the PCDL database and compared with data from the published literature. In this way, a total of 16 compounds were characterized, and their information and molecular formulas are summarized in Supplementary Table S3. These compounds were L-Tryptophan, 3-O-*p*-Coumaroylquinic acid, 4-O-*p*-Coumaroylquinic acid, Ophiopogonin K, 1-Borneol-beta-apisyl-beta-glucopyranoside, Protogracillin, 9,12,13-Trihydroxy-10E-octadecenoic acid, Ophiogenin 3-O-α-L-rhamnopyranosyl-(1→2)-β-D-glucopyranoside, 14-Hydroxy sprengerinin C, Ophiopogonanone E, Deacetyl ophiopojaponin A, Ophiopogonin P, Ophiopogonin D/Ophiopogonin D′, Mythylophiopogonanone B mononethyl ether, Methylophiopogonanone A, 1-Monopalmitin.

### 3.8. Network Pharmacological Analysis

**Figure 8:**
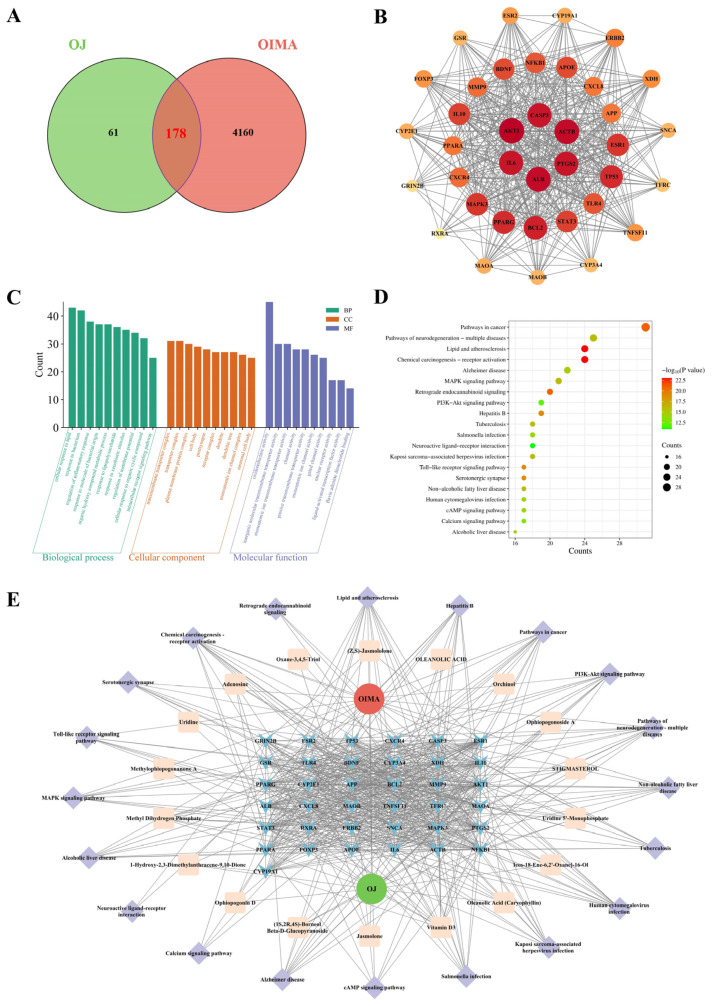
Network pharmacological analysis of OJ and muscle atrophy. (**A**) Venn diagram of common targets between OJ and obesity-induced muscle atrophy. (**B**) PPI network of core targets. (**C**) GO enrichment analysis of common targets. (**D**) KEGG pathway enrichment analysis of common targets. (**E**) “Disease-drug-core component-key target-KEGG pathway” interaction network.

To identify candidate molecular targets mediating the effects of OJ extract on obesity-induced muscle atrophy, we performed a network-pharmacology analysis. A total of 239 targets of OJ and 4338 targets of obesity-induced muscle atrophy were identified, with 178 common targets (Figure 8A). Core target screening of these 178 common targets using CentiScaPe 2.2 yielded 37 core targets (Figure 8B), among which ALB, IL6, AKT1, CASP3, ACTB, and PTGS2 ranked highest.

GO enrichment analysis was conducted across three categories: biological process (BP), cellular component (CC), and molecular function (MF). Figure 8C displays the top 10 enriched terms per GO category. GO biological process enrichment revealed that the top terms included “cellular response to lipid”, “regulation of inflammatory response”, and “regulation of membrane potential”. KEGG pathway enrichment revealed involvement of the “MAPK”, “PI3K-Akt”, and “Toll-like receptor “signaling pathway (Figure 8D). Finally, target information was categorized to construct a disease-drug-core component-key target-KEGG pathway interaction network (Figure 8E).

## 4. Discussion

Obesity, resulting from excessive energy intake and reduced physical activity, leads to abnormal fat accumulation in the body [22]. As adipose tissue loses its lipid storage capacity, circulating lipid levels increase, and excess lipids infiltrate skeletal muscle, where they accumulate within myofibers [23]. This ectopic lipid deposition induces lipotoxicity and mitochondrial dysfunction, ultimately contributing to insulin resistance and muscle atrophy [24]. Although exercise is an effective strategy for preventing and managing obesity-associated muscle atrophy, obese individuals often exhibit limited exercise capacity and poor adherence, making it difficult to maintain long-term physical activity and thereby diminishing the efficacy of exercise therapy as a primary intervention [25]. For these individuals, pharmacological treatments may serve as alternative or adjunctive strategies; however, synthetic drugs are frequently associated with undesirable side effects. In contrast, natural products, characterized by high safety and suitability for long-term consumption, offer potential for chronic regulation and preventive intervention. Moreover, they can be combined with exercise or rehabilitation therapies to overcome limitations caused by poor compliance. Therefore, the development of edible bioactive components with protective effects against obesity-induced muscle atrophy holds significant therapeutic and preventive value.

*Ophiopogon japonicus* is recognized as a superior-grade medicinal material in traditional practice with a favorable safety profile [17]. Recent pharmacological studies have reported that *O. japonicus* exhibits diverse biological activities, including anti-diabetic, anti-cancer, anti-inflammatory, antioxidant, anti-obesity, and cardiovascular protective effects [13,26]. Given its broad pharmacological properties and favorable safety, *O. japonicus* holds potential as a functional candidate for preventing obesity-associated muscle atrophy. In this study, we systematically investigated the effects and underlying mechanisms of OJ extract on obesity-induced muscle atrophy both in vivo and in vitro.

Chronic HFD feeding for inducing muscle atrophy in mice is a widely recognized method for studying the effects of prolonged obesity on skeletal muscle health [27]. Muscle atrophy, which is commonly associated with metabolic diseases such as obesity and diabetes, is characterized by diminished muscle strength and mass. In our behavioral tests, OJ extract enhanced grip strength and hanging time, and it also increased the weight and cross-sectional area of the gastrocnemius muscle, which is predominantly composed of fast-twitch (type II) fibers [28]. Reversing or preventing type II fiber atrophy represents an effective therapeutic strategy for muscle atrophy [29]. Moreover, muscles with high type II fiber ratios are prone to ubiquitin-proteasome system (UPS) activation, facilitating assessment of proteolytic pathways [30]. Atrogin-1 and MuRF1, muscle-specific E3 ubiquitin ligases and core UPS components, exhibit low baseline expression in healthy muscle but significant upregulation during atrophy, serving as molecular markers [30,31,32]. In this study, we found that treatment with OJ extract suppressed the expression of Atrogin-1 and MuRF1.

Meanwhile, FoxO3a, the master transcriptional regulator of Atrogin-1 and MuRF1 in muscle tissue, drives UPS-mediated proteolysis upon nuclear translocation following dephosphorylation [33]. This process is governed by the PI3K/AKT signaling pathway, which phosphorylates conserved FoxO3a sites to inhibit nuclear translocation and transcriptional activity [34]. Additionally, mTOR, a central kinase regulating cell growth, metabolism, and autophagy, functions as a critical “anabolic switch” in muscle atrophy [35]. It forms a cascade regulatory network with the PI3K/AKT signals to determine muscle protein synthesis-degradation balance. In mice with obesity-induced muscle atrophy, administration of OJ extract increased the phosphorylation of PI3K, AKT, mTOR, and FoxO3a in gastrocnemius tissues. Moreover, in C2C12 myotubes, treatment with OJ extract ameliorated PA-induced atrophic phenotypes through activation of the PI3K–AKT–mTOR/FoxO3a signaling pathway. These findings indicated that the anti-atrophic effects of OJ extract were mediated through regulation of the PI3K–AKT–mTOR/FoxO3a signaling pathway, which promotes protein synthesis while inhibiting protein degradation.

Lipid metabolic disorder is one of the core pathological mechanisms underlying obesity-induced skeletal muscle atrophy, involving multiple processes such as ectopic lipid deposition, imbalanced fatty acid metabolism, and lipotoxic stress [36,37]. Mice with obesity induced by an HFD exhibited significant increases in body weight, elevated FBG levels, impaired glucose tolerance, and insulin resistance, consistent with previous studies [38]. Following intervention with OJ extract, both body weights and blood glucose levels were markedly reduced in our obesity-induced muscle atrophy mice. The results of OGTT and ITT also indicated a restoration of insulin sensitivity by OJ extract in obese mice. Moreover, histological analysis of liver tissue revealed that OJ extract prevented obesity-induced hepatic steatosis, inflammatory infiltration, and ballooning degeneration. Additionally, serum levels of ALT, AST, TG, TCHO, and LDL-cholesterol were significantly decreased in OJ extract-administrated groups, suggesting that this extract has hepatoprotective effects by attenuating excessive lipid accumulation and preserving liver function. Further assessment of adipose tissue mass showed that OJ extract reduced the relative weights of iWAT and eWAT, without significantly affecting BAT, which may be related to the regulation of lipid synthesis and degradation balance by OJ extract.

We further analyzed changes in genes associated with lipid metabolic regulation, such as *CPT1b*, *SREBP-1c*, *DGAT2*, and *SCD1*, in both muscle tissues and C2C12 myotubes. Administration of OJ extract markedly enhanced *CPT1b* transcription and protein abundance, whereas the expression of *SREBP-1c*, *DGAT2*, and *SCD1* was markedly reduced in the gastrocnemius tissues and C2C12 myotubes. Increased expression of *CPT1b*, a key rate-limiting enzyme in fatty acid β-oxidation, promotes intracellular lipid catabolism [28]. Conversely, suppression of *SREBP-1c* and its downstream targets reduces triglyceride synthesis and ectopic lipid deposition [39]. Therefore, our results suggest that OJ extract may alleviate obesity-induced muscle atrophy through a multi-targeted mechanism involving lipid metabolism.

On the one hand, increasing CPT1b expression boosts mitochondrial β-oxidation, which diminishes the buildup of lipid intermediates such as ceramides and diacylglycerol. This reduction alleviates their suppressive influence on the PI3K/AKT/mTOR signaling pathway in myocytes [40]. On the other hand, the downregulation of *SREBP-1c* and its downstream genes further suppresses triglyceride synthesis and lipogenesis, establishing a dual regulatory pattern of “promotion of lipid degradation” and “inhibition of lipid synthesis” [41]. This synergistic effect not only improves lipid homeostasis in skeletal muscle but also restores AKT phosphorylation, activates mTOR-mediated protein synthesis, and inhibits FoxO3a nuclear translocation, ultimately leading to reduced expression of the E3 ubiquitin ligases [42]. Through coordinated regulation of metabolic and proteostatic pathways, OJ extract effectively counteracts the progression of muscle atrophy.

To validate the potential molecular mechanisms by which OJ extract acts on obesity-induced muscle atrophy, we performed a network pharmacological analysis. Our analysis identified multiple potential targets of OJ extract in obesity-induced muscle atrophy, with core nodes including AKT1, IL6, and CASP3. These genes participate in key biological functions, particularly those governing lipid metabolic regulation, inflammatory activity, and cellular survival. KEGG pathway enrichment revealed that signaling routes such as PI3K/Akt, MAPK, and TLR were strongly aligned with the pathways experimentally validated in our work, especially the PI3K–AKT–mTOR/FoxO3a axis, which has been repeatedly reported to be essential for maintaining muscle homeostasis [29,43,44]. UHPLC-MS/MS analysis identified ophiopogonin D and methylophiopogonanone A as the constituents of OJ extract. Ophiopogonin D has been reported to improve lipid metabolism, mitigate oxidative stress, and suppress inflammation in metabolic syndrome [45]. Meanwhile, methylophiopogonanone A has demonstrated cardioprotective and neuroprotective effects by activating the PI3K-Akt signaling pathway and suppressing apoptosis [46]. Collectively, these findings not only corroborate the in vivo and in vitro results obtained in the present study, but also provide direction for further investigation into the multi-targets and multi-pathways synergistic mechanisms of OJ extract. However, several limitations should be acknowledged. First, the key bioactive constituents of OJ extract, such as polysaccharides, saponins, and flavonoids, have not yet been fully identified or comparatively evaluated, which may limit its precise clinical application. As polysaccharides are also regarded as indispensable contributors to the biological activities of OJ, future studies will focus on isolating and purifying individual components such as polysaccharides, steroidal saponins, and flavonoids, followed by a systematic comparison of their differential biological effects. Second, the role of the PI3K-AKT-mTOR/FoxO3a signaling pathway was inferred mainly from phosphorylation changes without functional validation, and thus causal evidence is still lacking. Third, although the present findings are supported by animal and cell-based studies, well-designed clinical studies will be necessary to further assess the human applicability, therapeutic effectiveness, and safety profile of OJ extract.

**Figure 9:**
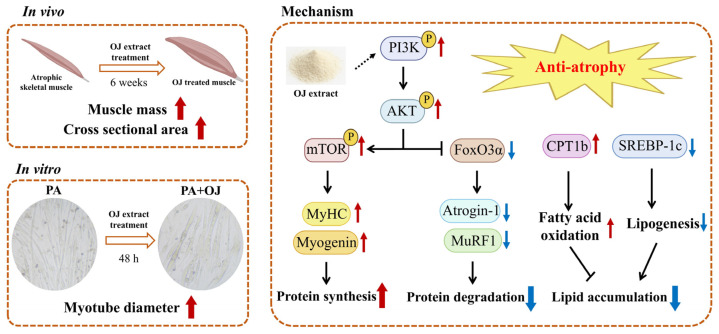
The potential mechanism of OJ extract for OIMA treatment (Red arrows, upregulation; blue arrows, downregulation).

In summary, the present study suggests that OJ extract improves the symptoms of obesity-induced muscle atrophy through regulation of the PI3K-AKT-mTOR/FoxO3a signaling pathway and reprogramming of lipid metabolism in muscle tissues of mice and C2C12 myotubes (Figure 9). These findings provide scientific evidence for the effects and mechanisms of OJ extract, support its potential as a therapeutic candidate for preventing and treating muscle atrophy, and highlight a novel therapeutic strategy for metabolic myopathies.

## 5. Conclusions

This study demonstrated that OJ extract alleviated obesity-induced muscle atrophy by activating the PI3K-AKT-mTOR/FoxO3a signaling pathway and regulating lipid metabolism. These findings indicate the therapeutic potential of OJ extract in managing obesity-related muscle atrophy and expand the pharmacological profile of OJ beyond its traditionally recognized activities.

## Data Availability

All datasets supporting the conclusions of this research are contained within the article and its Supplementary Materials.

## Supplementary Materials

The following supporting information can be downloaded at: https://www.mdpi.com/article/10.3390/nu17243946/s1, Figure S1: (A) Food intake. Masses of iWAT (B), eWAT (C) and BAT (D) were normalized to body weight (g). All data are presented as the mean ± SD of three independent experiments. The *p* values were defined as follows: ^###^ *p* < 0.001 vs. the CON group; ^*^ *p* < 0.05 and ^**^ *p* < 0.01 vs. the MOD group; NS = not significant (*p* ≥ 0.05). Figure S2: (A) C2C12 myotubes were treated with different concentrations of PA for 48 h. (B) Cell viability was evaluated in PA-stimulated C2C12 myotubes treated with different concentrations of OJ extract for 48 h. Quantification of MyHC positive area (C) and Myogenin positive area (D) in C2C12 myotubes was performed by immunofluorescence analysis. All data are presented as the mean ± SD of three independent experiments. The *p* values were defined as follows: ^###^ *p* < 0.001 vs. the CON group; ^*^ *p* < 0.05 and ^***^ *p* < 0.001 vs. the PA group. Table S1: The index for liver histopathological scoring. Table S2: The oligonucleotide primer sequences in RT-qPCR. Table S3: Chemical composition information of OJ extract identified using UHPLC-Q-TOF-MS/MS technology.
