# Supplementary material for: Ophiopogon japonicus Root Extract Attenuates Obesity-Induced Muscle Atrophy Through Regulation of the PI3K-AKT-mTOR/FoxO3a Signaling Pathway and Lipid Metabolism in Mice and C2C12 Myotubes
Source: Nutrients. 2025 Dec 17;17(24):3946. doi: 10.3390/nu17243946 (PMC12736110; doi:10.3390/nu17243946)
Supplement: Supplementary file 1 [file nutrients-17-03946-s001.zip › nutrients-4021360-supplementary.pdf]

Supplementary Material

Ophiopogon japonicus Root Extract Attenuates Obesity-Induced Muscle Atrophy Through Regulation of the PI3K-AKT-mTOR/FoxO3a Signaling Pathway and Lipid Metabolism in Mice and C2C12 Myotubes

Yang Wang [1], Haifeng Shao [1], Chenzi Lyu [1], Kyung Hee Park [1], Tran Khoa Nguyen [2], In Jun Yang [2], Hyo Won Jung [1,*] and Yong-Ki Park [1]

[1] Department of Herbology, College of Korean Medicine, Dongguk University, Gyeongju 38066, Republic of Korea; wy1997ere@163.com (Y.W.); shf326904@163.com (H.S.); lcz18435166779@163.com (C.L.); 2024126916a@gmail.com (K.H.P.); yongki@dongguk.ac.kr (Y.-K.P.)

[2] Department of Physiology, College of Korean Medicine, Dongguk University, Gyeongju 38066, Republic of Korea; trannguyen053@gmail.com (T.K.N.); injuny@gmail.com (I.J.Y.)

* Correspondence: tenzing2@hanmail.net; Tel.: +82-54-770-2367

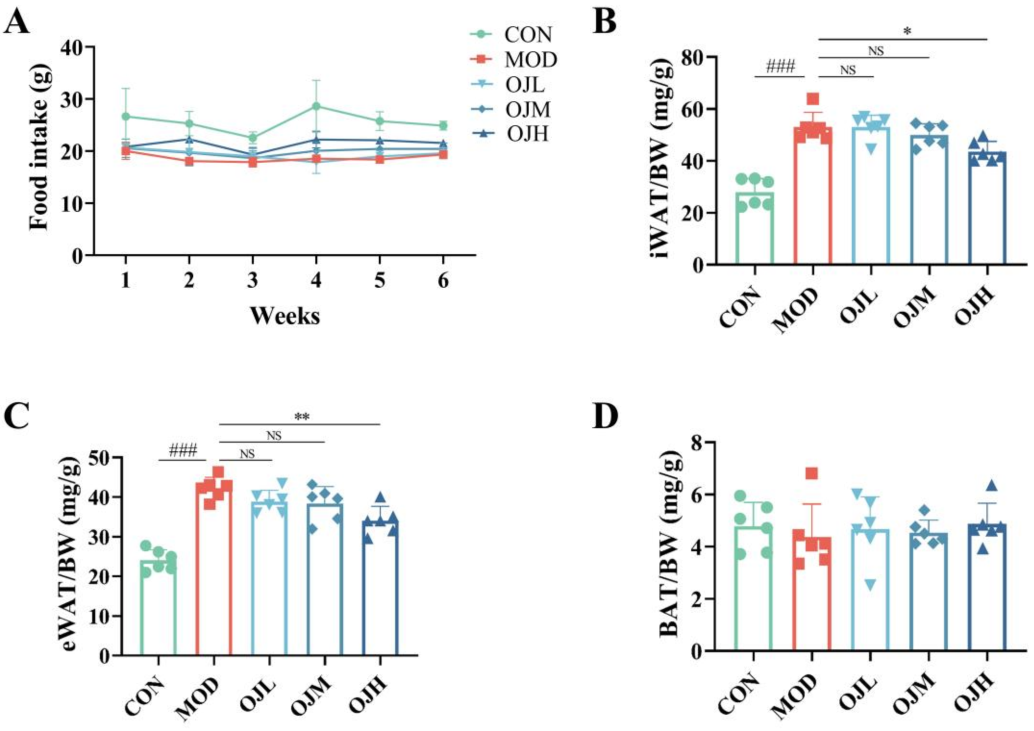

Figure S1. (A) Food intake. Masses of iWAT (B), eWAT (C) and BAT (D) were normalized to body weight (g). All data are presented as the mean ± SD of three independent experiments. The p values were defined as follows: $^{###}p < 0.001$ vs. the CON group; $^{*}p < 0.05$ and $^{**}p < 0.01$ vs. the MOD group; NS = not significant ($p \geq 0.05$).

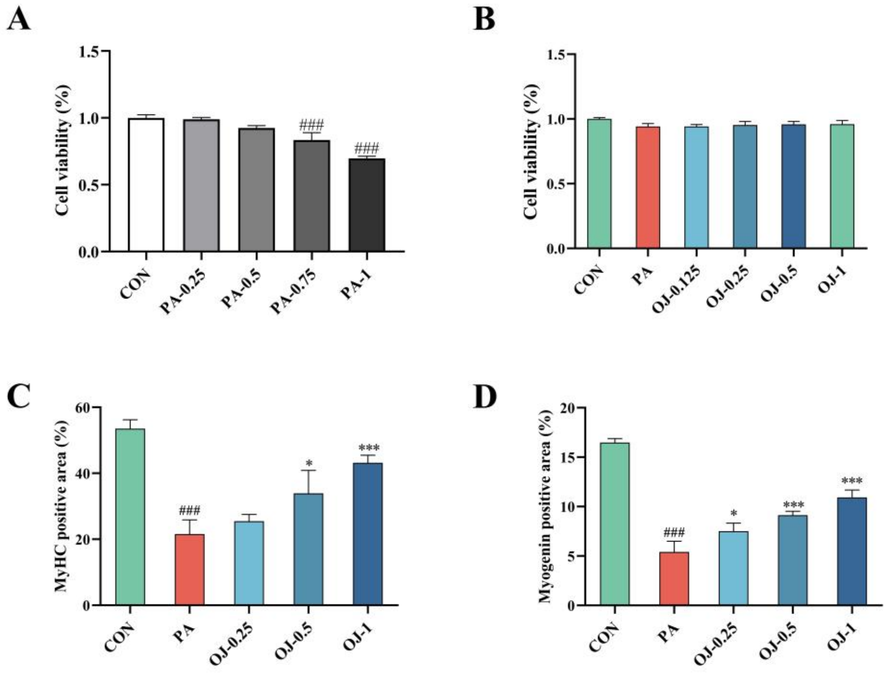

Figure S2. (A) C2C12 myotubes were treated with different concentrations of PA for 48 h. (B) Cell viability was evaluated in PA-stimulated C2C12 myotubes treated with different concentrations of OJ extract for 48 h. Quantification of MyHC positive area (C) and Myogenin positive area (D) in C2C12 myotubes was performed by immunofluorescence analysis. All data are presented as the mean ± SD of three independent experiments. The p values were defined as follows: $^{###}p < 0.001$ vs. the CON group; $^{*}p < 0.05$ and $^{***}p < 0.001$ vs. the PA group.

Table S1. The index for liver histopathological scoring.

Steatosis (%)	Lobular inflammation	Ballooning	Scores
Percentage ＜ 5	no foci	none	0
5 ≤ percentage ＜ 33	<2 foci	few balloon cells	1
33 ≤ percentage ＜ 66	2-4 foci	many balloon cells	2
66 ≤ percentage	>4 foci		3

Table S2. The oligonucleotide primer sequences in RT-qPCR.

Gene	Sequences 5′-3′ forward	Sequences 5′-3′ Reverse	Species
CPT1b	AGTGGCCTCACAGACTCCAG	GCCCATGTTGTACAGCTTCC	mice
SREBP-1c	AGATCCAGGTTTGAGGTGGG	ATCGCAAACAAGCTGACCTG	mice
DGAT2	GAAGATGTCTTGGAGGGCTG	CGCAGCGAAAACA AGAATAA	mice
SCD1	TTCTTGCGATACACTCTGGTGC	CGGGATTGAATGTTCTTGTCGT	mice
GAPDH	TGCAACCGGGAAGGAAATGA	GCCCAATACGACCAAATCAGA	mice

Table S3. Chemical composition information of OJ extract identified using UHPLC-Q-TOF-MS/MS technology.

NO.	Compound	Formula	Retention time (min)	Measured (m/z)	ESI ionization mode	ppm	MS^2
1	L-Tryptophan	$C_{11}H_{12}N_2O_2$	10.578	205.0971	$[M+H]^+$	0.96	188.0706, 170.0598, 159.0914, 146.0599, 132.0807, 118.0649
			10.578	203.0828	$[M-H]^-$	1.18	186.0560, 142.0666, 116.0503
2	3-O-p-Coumaroylquinic acid	$C_{16}H_{18}O_8$	13.621	339.1078	$[M+H]^+$	1.69	147.0445, 119.0493
			13.621	337.0939	$[M-H]^-$	1.75	191.0563, 163.0403, 119.0504
3	4-O-p-Coumaroylquinic acid	$C_{16}H_{18}O_8$	18.772	339.1072	$[M+H]^+$	0.68	199.1802, 147.0441, 119.0492
			18.772	337.0933	$[M-H]^-$	1.32	191.0562, 173.0457, 119.0503
4	Ophiopogonin K	$C_{51}H_{84}O_{25}$	31.751	1079.5299	$[M+H-H_2O]^+$	1.08	1043.5045, 881.4531, 719.3997, 573.3425, 411.2896
			31.751	1095.5230	$[M-H]^-$	-0.02	627.3013, 609.2895, 467.2130, 305.1586, 163.0605
5	1-Borneol-beta-apisyl-beta-glucopyranoside	$C_{21}H_{36}O_{10}$	34.800	466.2647	$[M+NH_4]^+$	1.38	295.1026, 137.1326, 115.0391
			34.800	493.2289	$[M+FA-H]^-$	1.25	447.2240, 315.1820, 161.0458, 101.0245
6	Protogracillin	$C_{51}H_{84}O_{23}$	38.926	1047.5379	$[M+H-H_2O]^+$	1.49	885.4842, 723.4315, 577.3733, 415.3203
			38.926	1063.5346	$[M -H]^-$	0.48	918.4838, 901.4818, 755.4182, 737.4092
7	9,12,13-Trihydroxy-10E-octadecenoic acid	$C_{18}H_{34}O_5$	46.895	348.2743	$[M+H-H_2O]^+$	0.85	295.2268, 277.2162, 195.1380, 155.1066, 109.1011
			46.895	329.2341	$[M-H]^-$	0.9	229.1446, 211.1342, 171.1025, 139.1130
8	Ophiogenin 3-O-α-L-rhamnopyranosyl-(1→2)-β-D-glucopyranoside	$C_{39}H_{62}O_{14}$	53.172	799.4128	$[M+FA-H]^-$	0.64	753.4071, 607.3515, 205.0724, 101.0240
9	14-Hydroxy sprengerinin C	$C_{44}H_{70}O_{17}$	57.903	869.4544	$[M-H]^-$	0.32	737.4197, 289.0887, 101.0260
10	Ophiopogonanone E	$C_{19}H_{20}O_7$	58.278	361.1286	$[M+H]^+$	0.93	237.0759, 137.0598, 109.0644

			58.278	359.1137	[M -H]-	0.18	344.0901, 208.0378, 169.0504, 154.0271, 124.0164
11	Deacetyl ophiopojaponin A	$C_{46}H_{72}O_{18}$	63.570	911.4647	[M -H]-	0.32	869.45417, 805.9860, 738.4142, 149.0463
12	Ophiopogonin P	$C_{41}H_{64}O_{14}$	64.601	781.4368	[M+H]+	-0.06	395.2941, 251.1795, 189.0756, 129.0548
			64.601	825.4277	[M+FA-H]-	-1.2	779.4216, 737.4114, 719.4010
13	Ophiopogonin D/Ophiopogonin D'	$C_{44}H_{70}O_{16}$	67.130	899.4649	[M+FA-H]-	0.32	853.4593, 721.4152, 575.3586, 145.0507, 103.0399
14	Mythylophiopogonanone B mononethyl ether	$C_{20}H_{22}O_5$	69.238	343.1552	[M+H]+	2.04	221.0810, 121.0650
			69.238	341.1395	[M -H]-	0.75	326.1147, 267.1385, 193.0510, 149.0604,109.0300
15	Methylophiopogonanone A	$C_{19}H_{18}O_6$	69.812	343.118	[M+H]+	3.76	207.0645, 135.0436
			69.805	341.103	[M -H]-	-0.18	206.0571, 178.0634, 150.1682
16	1-Monopalmitin	$C_{19}H_{38}O_4$	82.723	331.2850	[M+H]+	1.82	313.2738, 257.2476, 239.2370
			82.723	375.2753	[M+FA-H]-	-0.3	334.6573, 241.7943, 239.1598
